# Alternative academic approaches for testing homologous recombination deficiency in ovarian cancer in the MITO16A/MaNGO-OV2 trial

**DOI:** 10.1016/j.esmoop.2022.100585

**Published:** 2022-09-23

**Authors:** E.D. Capoluongo, B. Pellegrino, L. Arenare, D. Califano, G. Scambia, L. Beltrame, V. Serra, G.L. Scaglione, A. Spina, S.C. Cecere, R. De Cecio, N. Normanno, N. Colombo, D. Lorusso, D. Russo, C. Nardelli, M. D’Incalci, A. Llop-Guevara, C. Pisano, G. Baldassarre, D. Mezzanzanica, G. Artioli, M. Setaro, G. Tasca, C. Roma, N. Campanini, S. Cinieri, A. Sergi, A. Musolino, F. Perrone, P. Chiodini, S. Marchini, S. Pignata

**Affiliations:** 1 Department of Molecular Medicine and Medical Biotechnology, Università degli Studi di Napoli Federico II, Naples; 2 Azienda Ospedaliera per L'Emergenza, Cannizzaro, Catania; 3 Department of Medicine and Surgery, University of Parma, Parma; 4 Medical Oncology and Breast Unit, University Hospital of Parma, Parma; 5 Gruppo Oncologico Italiano di Ricerca Clinica (GOIRC), Parma; 6 Clinical Trial Unit, Istituto Nazionale Tumori, IRCCS, Fondazione G. Pascale, Naples; 7 Microenvironment Molecular Targets Unit, Istituto Nazionale Tumori IRCCS – Fondazione G. Pascale, Naples; 8 Department of Women and Child Health, Division of Gynecologic Oncology, Fondazione Policlinico Universitario A. Gemelli IRCCS, Rome; 9 Department of Life Science and Public Health, Catholic University of Sacred Heart Largo Agostino Gemelli, Rome; 10 Molecular Pharmacology laboratory., Group of Cancer Pharmacology IRCCS Humanitas Research Hospital, Rozzano, Italy; 11 Vall d’Hebron Institute of Oncology, Barcelona, Spain; 12 Advanced Biotechnology, Università Federico II-CEINGE, Naples; 13 IDI-IRCSS, Rome; 14 Uro-Gynecologic Oncology Unit, Istituto Nazionale Tumori IRCCS Fondazione G. Pascale, Naples; 15 Pathology Unit, Istituto Nazionale Tumori ’Fondazione Giovanni Pascale’, IRCCS, Napoli; 16 Cell Biology and Biotherapy Unit, Istituto Nazionale Tumori ’Fondazione Giovanni Pascale’, IRCCS, Napoli; 17 University of Milan-Bicocca and European Institute of Oncology IRCCS, Milan; 18 Department of Biomedical Sciences, Humanitas University, Pieve Emanuele, Milan; 19 Molecular Oncology Unit, Centro di Riferimento Oncologico di Aviano (CRO), IRCCS, National Cancer Institute, Aviano; 20 Molecular Therapies Unit, Department of Research, Fondazione IRCCS Istituto Nazionale dei Tumori, Milan; 21 Oncologia Medica, ULSS2 Marca Trevigiana, Treviso; 22 Division of Oncology 2, Istituto Oncologico Veneto IRCCS, Padova; 23 Unit of Pathological Anatomy, Department of Medicine and Surgery, University Hospital of Parma, Parma; 24 Oncologia Medica, Ospedale Senatore Antonio Perrino, Brindisi; 25 Department of Electronics, Information and Bioengineering, Politecnico di Milano, Milan; 26 Department of Mental Health and Public Medicine, Section of Statistics, Università degli Studi della Campania Luigi Vanvitelli, Napoli, Italy

**Keywords:** ovarian cancer, molecular testing, HRR, HRD, Myriad

## Abstract

**Background:**

The detection of homologous recombination deficiency (HRD) can identify patients who are more responsive to platinum and poly ADP ribose polymerase inhibitors (PARPi). MyChoice CDx (Myriad) is the most used HRD test in ovarian cancer (OC). However, some limitations of commercial tests exist, because of the high rate of inconclusive results, costs, and the impossibility of evaluating functional resistance mechanisms.

**Patients and methods:**

Two academic genomic tests and a functional assay, the RAD51 foci, were evaluated to detect HRD. One hundred patients with high-grade OC enrolled in the MITO16A/MaNGO-OV2 trial and treated with first-line therapy with carboplatin, paclitaxel, and bevacizumab were analyzed.

**Results:**

The failure rate of the two genomic assays was 2%. The sensitivity in detecting HRD when compared with Myriad was 98.1% and 90.6%, respectively. The agreement rate with Myriad was 0.92 and 0.87, with a Cohen’s κ coefficient corresponding to 0.84 and 0.74, respectively. For the RAD51 foci assay, the failure rate was 30%. When the test was successful, discordant results for deficient and proficient tumors were observed, and additional HRD patients were identified compared to Myriad; sensitivity was 82.9%, agreement rate was 0.65, and Cohen’s κ coefficient was 0.18. The HRD detected by genomic assays and residual tumor at primary surgery and stage was correlated with progression-free survival at multivariate analysis.

**Conclusions:**

Results suggest the feasibility of academic tests for assessing HRD status that show robust concordance with Myriad and correlation with clinical outcome. The contribution of the functional information related to the RAD51 foci test to the genomic data needs further investigation.

## Highlights

• Deficiency in homologous recombination repair of DNA generates genomic instability and permanent genomic changes.
• HRD status is fundamental for identifying OC patients suitable for platinum and PARPi treatment.
• HRD testing is considered a topic with urgent need for improvement, going beyond those available commercially.
• Within this study, two academic genomic tests and a functional assay, the RAD51 foci, were evaluated to detect HRD.
• Our tests compare favorably with the reference Myriad assay and correlate with the outcome of high-grade OC patients.

## Introduction

Ovarian cancer (OC) ranks first in mortality rate among all gynecological cancers due to the usually late diagnosis and the development of resistance.^1^^,^^2^ The standard first-line therapy is represented by carboplatin + paclitaxel; however, in recent years, the increasing understanding of the biology of OC has led to the introduction of new biological compounds in the therapeutic armamentarium. In particular, bevacizumab in combination regimens and maintenance after chemotherapy is approved in the first-line setting and recurrent disease; more recently, poly ADP ribose polymerase inhibitors (PARPi) have been approved for maintenance therapy after first-line chemotherapy (olaparib, niraparib) and in platinum-sensitive recurrences (olaparib, niraparib, rucaparib).^3^, ^4^, ^5^, ^6^, ^7^, ^8^, ^9^

The targets of PARPi are the PARP1/2 enzymes, which play a major role in the DNA repair system.^10^, ^11^, ^12^ Indeed, the homologous recombination repair (HRR) of DNA is a key pathway that repairs DNA double-strand breaks. Deficiency in HRR (HRD) generates genomic instability and permanent genomic changes, with specific patterns (‘genomic scars’). From a clinical perspective, displaying HRD has been correlated to better response to platinum derivatives and PARPi with higher susceptibility to cell death induced by these molecules.^10^, ^11^, ^12^ For instance, in the PRIMA and PAOLA1 trials, patients with HRD due to *BRCA1/2* mutations are more likely to respond to PARPi than those with wild-type (WT) *BRCA1/2*.^8^^,^^9^ In addition, patients not mutated in *BRCA* who resulted in HRD at genomic tests respond to PARPi more frequently than homologous recombinant proficient (HRP) patients. In the PAOLA 1 trial, the HRD test identified a population of HRP patients who do not benefit at all from the combination of olaparib and bevacizumab.^9^ Therefore, the identification of HRD is crucial to patient selection for PARPi. The first commercially available HRD assay was the myChoice CDx (Myriad; Myriad Genetics, Salt Lake City, UT), which detects *BRCA1* and *BRCA2* mutations along with the assessment of genomic instability combining the results of three molecular parameters: loss of heterozygosity, telomeric-allelic imbalance, and large-scale state transitions.^13^ This test is now considered the standard after the results of the PRIMA and PAOLA1 trials that used it to determine HRD status in the first-line setting.^14^

However, some limitations of this and other available tests exist and include the proportion of samples returned with inconclusive results, false-negative results, and high cost.^9^^,^^11^

Moreover, the identification of scars is not useful to detect the mechanisms of resistance that develop during PARPi therapy and functional information of the pathway’s activity.^15^ Consequently, newer approaches are required to improve the management of patients eligible for PARPi.^16^ To this end, the detection of the HRR protein RAD51, forming nuclear foci after DNA damage, has been investigated as a surrogate of HRR functionality and resulted in being correlated with PARPi resistance.^17^ The feasibility of the RAD51 assay in routine formalin-fixed paraffin-embedded (FFPE) tumor samples has been recently demonstrated, especially in identifying patients with breast cancer that might be sensitive to PARPi.^18^^,^^19^ A low RAD51 score was also related to PARPi response in patients with prostate cancer, primary triple-negative breast cancer (TNBC), and OC.^20^, ^21^, ^22^

We recently published the MITO16A/MaNGO-OV2 trial results, which investigated clinical and biological prognostic factors for advanced OC patients on first-line therapy with carboplatin, paclitaxel, and bevacizumab.^23^ Preliminary biomarker data have been recently published.^24^

Here, we report the comparison between three different molecular strategies for evaluating HRR status, developed in academic laboratories, and the Myriad test within a sub-population of untreated OC patients enrolled in the MITO16A/MaNGO-OV2 trial.

## Patients and methods

### Study design

The experiments were conducted in four academic laboratories with specific backgrounds in the evaluation of the homologous recombination pathway in OC [Istituto Nazionale Tumori IRCCS, Naples; Humanitas Research Hospital, Rozzano; Federico II University Hospital, Naples and Cannizzaro Hospital, Catania; Vall d'Hebron Institute of Oncology (VHIO), Barcelona, and University Hospital of Parma, Parma].

MITO16A/MaNGO-OV2 is a multicenter study coordinated by the Istituto Nazionale Tumori in Naples, Italy. From the whole population of the MITO16A/MaNGO-OV2 trial, we randomly selected 100 patients with high-grade serous and endometrioid cancer and enough material to allow all the assays (Myriad, genomic assays, RAD51 test) to be done on the same specimen. In order to evaluate the representativeness of the enrolled population, the characteristics of the identified patients were compared with those of the overall population of the MITO16A/MaNGO-OV2 trial, in terms of age, Eastern Cooperative Oncology Group performance status (ECOG PS), residual disease, and International Federation of Gynaecology and Obstetrics (FIGO) stage; progression-free survival (PFS) and overall survival (OS) in the two populations were also described. The 100 randomly selected specimens were tested by Myriad, LAB1 (Humanitas Research Hospital), LAB2 (Federico II University and Cannizzaro Hospital), and LAB3 RAD51 assay (VHIO and University Hospital of Parma). LAB1, LAB2, and LAB3 (see following section for detailed information on the assays and sample collection) evaluated their own tests blinded to the other labs’ results, Myriad test, and clinical information. Further details on patients and treatments of the MITO16A/MaNGO-OV2 trial and on specimen collection and handling are reported in the Supplementary Methods, available at https://doi.org/10.1016/j.esmoop.2022.100585. The institutional review boards of the involved institutions have approved the study design. All patients provided informed consent to the use of their data for research purposes before enrolment.

### Investigational assays

#### LAB1

A customized capture sequencing library was designed from a modification of a commercially available kit (Agilent OneSeq Constitutional Panel, Agilent, Santa Clara, CA), spanning 12 Mbp of structural genomic regions (‘backbone’) plus the target region.

Pre-processing and analysis followed current best practices (https://gatk.broadinstitute.org/hc/en-us). The complete procedure is available in the Supplementary Methods, available at https://doi.org/10.1016/j.esmoop.2022.100585.

Somatic variants in the *BRCA1/2* genes were extracted from the resulting data after the variant calling step, plus adjustments for purity, ploidy, and allele copy number. The Cancer Genome Interpreter^25^^,^^26^ and BRCA Exchange^27^ databases were used to remove benign, likely benign, passenger (i.e. variants predicted *in silico* not to be tumor drivers) variants and variants of unknown significance. The complete procedure for variant calling is described in the Supplementary Methods, available at https://doi.org/10.1016/j.esmoop.2022.100585.

##### HRD score calculation

The HRD score was calculated using an in-house developed Python script (see Supplementary Methods, available at https://doi.org/10.1016/j.esmoop.2022.100585), and the resulting HRD scores were then subject to a threshold (calculated as the fifth percentile from an internal dataset with *BRCA1/2* somatic and germline variants): HRD score >42 indicates probable HR deficiency; HR score <42 indicates probable HR proficiency. In addition, if a sample was indicated as HR proficient but presented a mutation in *BRCA1* or *BRCA2*, it was considered HR deficient; if a sample was indicated as HR proficient and had no other mutations, it was considered HR proficient.

#### LAB2

DNA libraries for Illumina sequencing were prepared using the KAPA HyperPlus kit (Roche Sequencing Solutions, Pleasanton, CA). The complete procedure is reported in the Supplementary Methods, available at https://doi.org/10.1016/j.esmoop.2022.100585.

Overall statistics for each next-generation sequencing (NGS) run were evaluated with the latest available version of the Illumina Run Manager software installed on the instrument. Further details are available in the Supplementary Methods, available at https://doi.org/10.1016/j.esmoop.2022.100585.

To investigate our tumor samples’ *BRCA* status (HRR assessment), we used the TruSight™ Tumor 170 kit (namely TS170; Illumina, San Diego, CA), an enrichment-based targeted panel. NGS was carried out following the manufacturer’s instructions; further details are reported in Supplementary Methods, available at https://doi.org/10.1016/j.esmoop.2022.100585.

##### HRD score calculation

HRD scores were calculated using whole-genome sequencing (WGS) data at low coverage (0.4-0.8×) for each sample using six different integrated models encompassing variable sliding windows spanning 5-1000 Kb. The HRD score was then estimated by measuring the level of agreement in the segmentation profiles of each sample and was independently calculated without considering the *BRCA* status. In more detail, to investigate the HRD status of our 100 samples, a LAB2 bioinformatics pipeline was developed, tested, and validated on a set of 60 somatic samples, 30 HRP, and 30 HRD that were previously screened by NGS for *BRCA1*/*2* alterations. Overall, to calculate HRD score from shallow WGS, our bioinformatics pipeline was developed as follows: binary aligned map (BAM) files are used as input and passed to QDNAseq R script (available at: https://bioconductor.org/packages/release/bioc/html/QDNAseq.html).

Once the files are processed, the aforementioned script returns a plain text file (formatted in Control-FREEC’s output) that is used as input for the shallow-HRD R script (available at: https://github.com/aeeckhou/shallowHRD).^28^ Our method uses a set of six different windows size bin (while segmenting the chromosomal coverage data in the QDNAseq script) before launching the shallow-HRD script, thus increasing the performances of the entire process. To briefly describe the methods proposed herein by LAB2, please refer to Supplementary Figure S1, available at https://doi.org/10.1016/j.esmoop.2022.100585 showing the workflows for the ‘standard’ and ‘LAB2’ protocols. Specifically, the steps in the red boxes (LAB2 side) are solely the ones we have tuned and designed in our bioinformatics pipeline.

#### LAB3

Immunofluorescence was carried out on 3-μm thick FFPE tumor sections as described before^18^ at the Vall d’Hebron Institute of Oncology (VHIO), where the test was initially validated in TNBC. The results were read both at VHIO and at the University of Parma. Antibodies used for immunofluorescence are reported in the Supplementary Methods, available at https://doi.org/10.1016/j.esmoop.2022.100585.

##### HRD score calculation

Scoring of biomarkers was carried out twice by the same investigator using the identical model of a microscope (Nikon TiE, ×60-immersion oil lens)—first at VHIO and then at the University of Parma. The RAD51 score represents the percentage of geminin-positive cells with five or more RAD51 nuclear foci. We evaluated, in parallel to RAD51/GMN staining and scoring, γH2AX/GMN staining and scoring in all samples as a quality check. The presence of γH2AX foci indicates the presence of double-strand break DNA damage, which is the prerequisite for HRR activation and RAD51 foci formation. In order to avoid false RAD51 low cases, samples with low γH2AX (<25% of geminin-positive cells with γH2AX foci) or with <40 geminin-positive cells were not included in the analyses due to insufficient endogenous DNA damage or tumor cells in the S/G_2_ phase of the cell cycle, respectively. A pre-defined cut-off of 10% for the RAD51 score was used to qualify tumors as HRD (≤10%) or HRP (>10%).^29^ A total of 100 geminin-positive cells from at least three representative areas of each sample were analyzed.

### Statistical analysis

Baseline characteristics of patients were described using median and interquartile range for continuous variables and frequencies and percentages for qualitative variables.

To evaluate the concordance of HRD academic tests with the commercial Myriad myChoice, it was calculated that a sample size of 89 patients would have resulted in a two-sided 95% confidence interval (CI) with a width of 0.25 considering a Cohen’s κ value of 0.80 and a standard deviation of K value of 0.60. The final sample size was increased to 100 patients considering the event of inconclusive tests.

The agreement and disagreement rates with the Myriad test were calculated for each academic test on the complete cases. The concordance index was measured using Cohen’s κ statistic with 95% CI. The K statistic was interpreted as <0 indicating no agreement, 0.00-0.20 as slight, 0.21-0.40 as fair, 0.41-0.60 as moderate, 0.61-0.80 as substantial, and 0.81-1.00 as an almost perfect agreement. For *BRCA* status, an agreement was calculated considering the number of mutations and the finding of the same mutations by the different tests.

The prognostic value of each HRR test (Myriad, LAB1, LAB2, LAB3) was investigated in terms of PFS, OS, and response rate (RR) by HRR status (HRD versus HRP). PFS was defined as the time from registration to documented progression according to RECIST criteria, death from any cause, or last follow-up date. OS was defined as the time from randomization to death from any cause or last information on the vital status.

Survival curves were calculated using the Kaplan–Meier method and compared by a log-rank test. Hazard ratios (HRs) were estimated by the Cox regression model. In the multivariable models, the following covariates were added: age (as category <65 versus ≥65 years), ECOG PS (0 versus 1-2), residual disease (none; ≤1 cm; >1 cm/not operated), and FIGO stage (III versus IV). The RR by investigator-assessed RECIST 1.1 was defined as the proportion of patients who had a complete or partial response. The exact CI of the proportion of respondent patients was calculated with the Clopper–Pearson method. All the analyses were carried out with STATA 14 MP (StataCorp. 2015. Stata Statistical Software: Release 14. College Station, TX: StataCorp LP.)

## Results

Baseline characteristics of the patients in the analysis and comparison with the MITO16A population are presented in Table 1.

**Table 1:** Baseline characteristics of patients (*n* = 100) and comparison with the MITO16A/MaNGO-OV2 population (*n* = 398)

Features	HRR sample, *n* (%)	MITO16A population, *n* (%)
Age (years), median (IQR)	57.8 (50.0-66.3)	59.2 (49.9-66.5)
Age category:		
• <65 years	70 (70.0)	278 (70.0)
• ≥65 years	30 (30.0)	120 (30.0)
ECOG performance status:		
• 0	80 (80.0)	315 (79.0)
• 1-2	20 (20.0)	83 (21.0)
Residual disease:		
• None	36 (36.0)	153 (38.0)
• ≤1 cm	25 (25.0)	72 (18.0)
• >1 cm/not operated	39 (39.0)	173 (43.0)
FIGO stage:		
• IIIB	9 (9.0)	36 (9.0)
• IIIC	72 (72.0)	275 (69.0)
• IV	19 (19.0)	87 (22.0)
Histology:		
• Serous grade 3	98 (98.0)	333 (84.0)
• Low-grade serous	0 (0)	13 (3.0)
• Endometrioid grade 3	2 (2.0)	9 (2.0)
• Clear cell	0 (0)	11 (3.0)
• Mucinous	0 (0)	3 (1.0)
• Mixed	0 (0)	4 (1.0)
• Other	0 (0)	25 (6.0)

ECOG, Eastern Cooperative Oncology Group; FIGO, International Federation of Gynaecology and Obstetrics (Fédération Internationale de Gynécologie et d’Obstétrique); HRR, homologous recombination repair; IQR, interquartile range.

Out of the 100 patients, 64 (64%) were eligible for RECIST response and 46 (72%) had complete (31, 48%) or partial (15, 23%) response. PFS was evaluated in all patients, with 80 progression events and a median PFS of 19.2 months (95% CI 16.0-21.9 months). Median OS was 40.7 months (95% CI 34.8-42.0 months), with 42 deaths.

A multivariate analysis on PFS, including age, stage, residual disease, and ECOG PS, was independently associated with prognosis (data not shown).

Supplementary Figure S2, available at https://doi.org/10.1016/j.esmoop.2022.100585, shows the number of patients assessable by each test for concordance along with the reason for test failure, as detailed in the following section.

### HRR status and *BRCA* status assessed by the Myriad test

Out of 100 samples analyzed by Myriad, 4 (4%) samples were inconclusive for HRR status because it was impossible to calculate the genomic instability score; 2 (2%) samples failed due to tissue quality. Consequently, Myriad HRR status was available for 94 (94%) samples, 53 of which (56%) were HRD and 41 (44%), HRP.

*BRCA1/2* status was available for 98 samples (98%) because two samples failed due to tissue quality. A total of 66 patients out of 98 analyzed (67%) were identified with Myriad as *BRCA* WT and 32 (33%) as *BRCA* mutated (24 *BRCA1* and 8 *BRCA2* mutated).

### HRD score and *BRCA* concordance between LAB1 assay and Myriad test

LAB1 HRD test was feasible in 97 out of 100 samples (97%), including one that failed the Myriad test. With the LAB1 test, 61 (63%) patients were deficient and 36 (37%) proficient for HRR.

For 92 samples, both LAB1 and Myriad tests were available (Table 2). Sensitivity of LAB 1 was 98.1% (95% CI 90.1% to 100%) and specificity was 84.2% (95% CI 68.7% to 94%). The agreement rate was equal to 0.92 (95% CI 0.87-0.98); Cohen’s κ coefficient corresponded to 0.84 (95% CI 0.72-0.96) (Table 3).

**Table 2:** Distribution of samples for HRR status, data for experimental assays and Myriad test

	Myriad, *n* (%)			Total (*n*)
	HRD	HRP	Missing	
LAB1:				
HRD	53 (64.4)	6 (15.4)	2 (33.3)	61
HRP	1 (1.8)	32 (82.1)	3 (50.0)	36
Missing	1 (1.8)	1 (2.6)	1 (16.7)	3
Total	55	39	6	100
LAB2:				
HRD	48 (87.3)	6 (15.4)	2 (33.3)	56
HRP	5 (9.1)	33 (84.6)	3 (50.0)	41
Missing	2 (3.6)	0 (0.0)	1 (16.7)	3
Total	55	39	6	100
LAB3:				
HRD	34 (61.8)	16 (41.0)	3 (50.0)	53
HRP	7 (12.7)	8 (20.5)	1 (16.7)	16
Missing	14 (25.5)	15 (38.5)	2 (33.3)	31
Total	55	39	6	100

HRD, homologous recombination deficiency; HRP, homologous recombination proficiency; HRR, homologous recombination repair; LAB, laboratory.

**Table 3:** Analysis of concordance of HRR status, data for experimental assays and Myriad test

	LAB1 (95% CI)	LAB2 (95% CI)	LAB3 (95% CI)
Number of samples evaluated for HRR	92	92	65
Agreement rate	0.92 (0.87-0.98)	0.87 (0.81-0.94)	0.65 (0.53-0.77)
Cohen κ	0.84 (0.72-0.96)	0.74 (0.60-0.88)	0.18 (−0.07 to 0.42)

CI, confidence interval; HRR, homologous recombination repair; LAB, laboratory.

All 100 samples in LAB1 were tested for *BRCA*, mutated in 29 (29%) cases, including 1 case that resulted in WT at the Myriad test.

Analysis of concordance of *BRCA* mutational status of Myriad and LAB1 was carried out on 98 samples. Out of 32 cases mutated at the Myriad test, 6 were WT and 1 had a different mutation at the LAB1 test. The agreement rate was equal to 0.92 (95% CI 0.86-0.97); Cohen’s κ coefficient was equal to 0.81 (95% CI 0.67-0.94).

### HRD score and *BRCA* concordance between LAB2 assay and Myriad test

Out of 100 samples, the HRD LAB2 test was feasible in 97 (97%), including one that failed the Myriad test. With the LAB2 test, 56 (58 %) patients were deficient and 41 (42 %) were proficient in HRR. For 92 samples, both LAB2 and Myriad tests were available (Table 2). Sensitivity for LAB2 was 90.6% (95% CI 79.3% to 96.9%) and specificity was 84.6% (95% CI 84.6% to 94.1%). The agreement rate was equal to 0.87 (95% CI 0.81-0.94); Cohen’s κ coefficient corresponded to 0.74 (95% CI 0.60-0.88) (Table 3).

Somatic *BRCA* mutation detection (evaluated by the software Illumina Pierian) was feasible by LAB2 in 88 samples since the remaining 12 out of 100 did not meet the quality criteria for this assay. *BRCA* variants were found in 45 (51%) cases, including 25 non-*BRCA* mutant cases at the Myriad test. Analysis of concordance of *BRCA* mutational status between Myriad and LAB2 was carried out in 86 samples. Out of 28 cases mutated at the Myriad test, 9 were WT and 4 had a different mutation at the LAB2 test, as evaluated by the TS170 assay. In 25 out of 58 patients who resulted as WT with Myriad, a mutation was found in the LAB2 test. The agreement rate was equal to 0.56 (95% CI 0.44-0.67); Cohen’s κ coefficient was equal to 0.09 (95% CI −0.11 to 0.30).

### Concordance between LAB3 assay and HRD score by Myriad

Out of 100 samples, 1 case was excluded because the slides did not contain tumor cells. In total, 99 samples were tested; 10 samples did not pass the quality check due to the lack of tumor cells in the S/G_2_ phase of the cell cycle (<40 geminin-positive cells); 20 samples exhibited insufficient endogenous DNA damage (<25% of geminin-positive cells with γH2AX foci).

Thus, out of 99 samples, the HRD LAB3 test was feasible in 69 (70%), including 2 that failed the Myriad test. According to the LAB3 test, 53 out of 69 cases (77%) were HR deficient, and 16 (23%) were HR proficient (Table 2). Sixty-five cases were available for the agreement analysis with both Myriad and LAB3 results. For LAB3, a sensitivity of 82.9% (95% CI 67.9% to 92.8%) and specificity of 33.3% (95% CI 15.6% to 55.3%) was found. The agreement rate was equal to 0.65 (95% CI 0.53-0.77); Cohen’s κ coefficient corresponded to 0.18 (95% CI: −0.07 to 0.42) (Table 3).

### Prognostic ability of HRD status

Table 4 shows PFS and OS in all patients according to each test and RR in those assessable by RECIST. RR in chemotherapy was higher in HRD compared to HRP cases for all tests, although the small numbers do not allow the statistical comparison.

**Table 4:** Outcomes by HRR status, data for experimental assays and Myriad test

	Myriad		LAB1		LAB2		LAB3	
	HRD	HRP	HRD	HRP	HRD	HRP	HRD	HRP
Eligible for RECIST assessment	59		63		62		47	
Response rate (95% CI)	82.4% (64.8% to 92.2%)	60% (38.8% to 78.0%)	84.2% (68.1% to 93.0%)	56.0% (35.3% to 74.8%)	75.8% (57.4% to 87.9%)	65.5% (45.7% to 81.0%)	90.9% (46.3% to 99.1%)	72.2 (54.7% to 84.8%)
Eligible for survival analysis	94		97		97		69	
Median PFS (months) (95% CI)	18.6 (12.0-22.3)	20.2 (17.0-25.3)	19.8 (16.3-24.2)	18.6 (12.0-22.6)	20.8 (16.3-27.5)	17.7 (12.0-22.1)	19.2 (15.8-22.1)	17.7 (9.9-25.1)
Median OS (months) (95% CI)	40.6 (27.3-NE)	41.1 (34.8-NE)	61 (37.9-NE)	39.7 (24.7-NE)	41.1 (34.8-42.0)	39.7 (24.7-NE)	37.9 (27.2-NE)	39.7 (17.9-NE)

HRD, homologous recombination deficiency; HRP, homologous recombination proficiency; HRR, homologous recombination repair; LAB, laboratory; NE, not estimated; PFS, progression-free survival; OS, overall survival.

Table 5 shows univariate and multivariate analysis, including HRR status in the prognostic model. Multivariate evaluation was carried out only on PFS due to the low number of events for OS. LAB2 results were prognostic in univariate analysis for PFS, while in the multivariate analysis, Myriad, LAB1 and LAB2 were correlated with PFS. LAB3 was not prognostic, probably due to the lower number of patients included.

**Table 5:** Univariate Cox regression models on progression-free survival and overall survival and multivariable Cox regression models only on progression-free survival

	PFS univariate			PFS multivariable^a^		OS univariate		
	Events	HR (95% CI)	*P* value	HR (95% CI)	*P* value	Events	HR (95% CI)	*P* value
Model 1: Myriad test:								
• HRD versus HRP	75	0.68 (0.43-1.08)	0.101	0.53 (0.33-0.86)	0.010	39	0.71 (0.38-1.34)	0.297
Model 2: LAB1 test:								
• HRD versus HRP	78	0.70 (0.44-1.10)	0.121	0.61 (0.38-0.98)	0.042	41	0.60 (0.33-1.12)	0.109
Model 3: LAB2 test:								
• HRD versus HRP	77	0.58 (0.37-0.91)	0.019	0.53 (0.33-0.85)	0.008	42	0.70 (0.38-1.29)	0.258
Model 4: LAB3 test:								
• HRD versus HRP	55	0.89 (0.49-1.64)	0.718	0.94 (0.50-1.76)	0.838	30	1.05 (0.46-2.38)	0.911

CI, confidence interval; HR, hazard ratio; HRD, homologous recombination deficiency; HRP, homologous recombination proficiency; LAB, laboratory; OS, overall survival; PFS, progression-free survival.

a Each multivariable model was adjusted for age (as category <65 versus ≥65 years). Eastern Cooperative Oncology Group performance status (0 versus 1-2), residual disease (none; ≤1cm; >1 cm/not operated), FIGO stage (III versus IV).

Supplementary Figures S3 and S4, available at https://doi.org/10.1016/j.esmoop.2022.100585, show the curves produced by the univariate analysis for all HR tests in PFS and OS, respectively.

### Combined analysis of Myriad and LAB3 test

The baseline characteristics of patients, PFS, and OS according to HRR status were comparable after the Myriad test or RAD51 assay HRD/HRP stratification (data not shown).

Combining the results of Myriad and RAD 51, 34 and 6 patients were classified as HRD or HRP in both tests, respectively. Five patients were found to be deficient in the Myriad test and proficient in the RAD51 test, and six patients were found to be proficient in the Myriad test and deficient in the RAD51 test. These four groups are too small and need to be explored prospectively. The curves are reported in Supplementary Figure S5, available at https://doi.org/10.1016/j.esmoop.2022.100585.

## Discussion

In the present study, two genomic tests of genetic instability carried out with different techniques and a functional assay, the RAD51 test, were evaluated to detect HRD and compared with Myriad, considered a reference standard. Of note, all assays were carried out on the same samples from patients enrolled in the MITO16A/MaNGO OV-2 trial with high-quality clinical data.^23^ All samples were collected before the initiation of chemotherapy, representing a pure picture of baseline molecular characteristics of untreated patients with advanced OC and allowing a correlation with the clinical outcome reported in the trial.

Overall, a high level of concordance between the two genomic approaches, namely LAB1 and LAB2, with the HRD status assessed with the Myriad test was found. This high concordance was paralleled with a very low failure rate, suggesting the feasibility of LAB1 and LAB2 assays. Interestingly, the failure rate was lower than that in previous studies, such as the PRIMA and PAOLA1 trials both for Myriad and LAB1 and LAB2 tests.^8^^,^^9^ This discrepancy can be likely attributed to differences in the preanalytical processing of samples that was centralized in the coordinating institution with a standardized procedure, as described previously.^30^ Given the potential feasibility of LAB1 and LAB2 genomic tests, it is possible to plan future studies to further investigate the use of these assays, for instance, with an ongoing prospective validation phase that is carried out in the ongoing MITO35a study, which evaluates treatment with PARPi in patients with WT *BRCA* status, in the same patient setting as that of the MITO16A/MaNGO OV-2 trial.^31^ Considering the LAB1 test, although the metrics used were similar to those reported by Telli et al.^13^—subsequently applied by Myriad myChoice test—there are some differences, and additional genomic information added in the validation phase might improve its predictive value and differentiate it from Myriad.

Interestingly, Cox analysis showed that LAB1, LAB2, and Myriad data in a multivariate model including the stronger prognostic variables, such as residual disease and stage, can separate patients at different risk of disease progression according to HRR status, confirming that HRR is related to the outcome to platinum-based therapy.

Of note, a lower concordance for *BRCA* status was found comparing Myriad with LAB1 and LAB2 tests. In particular, based on the current results, it has been decided for LAB2 to use in the prospective phase a different *BRCA* test based on a fully integrated bioinformatics workflow, SOPHiA DDM™, as a replacement for TST170 in the Illumina TruSight™ Tumor 170 panel. The TS170 tool has demonstrated its high limits, mainly referable to three important factors: (i) the on-bench industriousness of the method; (ii) costs of the entire chemistry; (iii) limitations of the software Pierian DX IVD that is still not ready for its implementation in clinical practice, especially due to the lack of capability to provide ready-to-use sequencing data. Our data suggest that high-quality programs for somatic *BRCA* testing are urgently needed to compare different software and methods.

As discussed, the assessment of functional activity of the HRR pathway would provide very relevant information for the selection of patients eligible for PARPi therapy in clinical practice. Indeed, the RAD51 functional test has already been proposed in breast and prostate cancer settings.^19^, ^20^, ^21^ In the present study, we used LAB3 testing to evaluate RAD51 functionality. A failure rate of 30% was reported for the RAD51 assay. We speculated that these suboptimal results, compared with the experience collected in breast cancer, can be due, at least in part, to the different quality of the paraffin-embedded samples between breast biopsy and ovarian surgical samples in terms of timing to and of fixation. We observed discordant results for deficient and proficient tumors when the test was successful. We analyzed tumor samples with inconclusive results at genomic assays in some cases. Interestingly, the RAD51 assay could also identify additional HRD patients compared with Myriad testing. However, due to the small number of patients, we were unable to firmly demonstrate that the RAD51 test can predict the response to platinum better. Similarly, the small sample size may have led to the lack of statistical significance for the prognostic ability of the combination of genomic and functional RAD51 testing. Of note, patients with HRP status at both genomic and RAD51 testing showed a trend toward a shorter PFS. Indeed, the possibility of combining a genomic and a functional test to improve the management of patients with OC is of great importance. However, this hypothesis is only speculative, given the limited sample size, and will be tested in the validation phase, during which sampling will be improved to minimize preanalytical issues.

Further analyses in the discordant cases are ongoing exploring specific gene mutation, gene expression, and micro RNA related to RAD51 pathway.

### Conclusion

Our analysis suggests the feasibility of different academic tests testing for HRD status, with a good concordance with the current standard, the Myriad assay. Prospective validation is ongoing, and other functional/epigenetic evaluations will soon be available, namely the targeted methylation assessment of HRR genes. In addition, as different metrics are currently under evaluation across different cancer types as predictive biomarkers, the availability of an academic test to assess the genome instability patterns makes assessment with other published genomic scars (e.g. AiCNA for breast cancer^32^) feasible in the near future, to expand the portfolio of tumor types in which the analysis of the genomic instability might help overcome the limitation of current approaches and improve the selection of the most suitable patients for therapies based on PARPi or other DNA-damaging agents.
