# Supplementary material for: Alternative academic approaches for testing homologous recombination deficiency in ovarian cancer in the MITO16A/MaNGO-OV2 trial
Source: ESMO Open. 2022 Sep 23;7(5):100585. doi: 10.1016/j.esmoop.2022.100585 (PMC9512829; doi:10.1016/j.esmoop.2022.100585)
Supplement: Supplementary_Methods [file mmc1.docx]

**Alternative academic approaches for testing homologous recombination deficiency in ovarian cancer in the MITO 16A/MaNGO-OV2 trial**

**SUPPLEMENTARY METHODS**

**Patients and treatments**

The MITO16A/MaNGO-OV2 trial has been reported elsewhere.^1^ In brief, the main enrolment criteria were as follows: FIGO stage IIIB–IV, previously untreated epithelial ovarian cancer, ECOG PS 0–2, adequate organ function and no prior major systemic disease. All patients in the MITO16A/MaNGO-OV2 trial received carboplatin and paclitaxel plus bevacizumab, followed by bevacizumab monotherapy, up to a maximum of 22 total cycles.

# **Specimen collection and handling**

# MITO16A/MaNGO-OV2 samples have been collected and processed in the coordinating center.^2^ A 5 µm section was cut from each FFPE block, stained with H&E, and reviewed by a trained gynecologic pathologist. In total, 100 samples of HGOC were selected for dedicated analyses. Histological sections were made from the paraffin blocks and sent to LAB3 or Myriad.

# According to the manufacturer's instructions, high-quality genomic DNA (gDNA) purification was carried out from two cores of paraffin-embedded tissues using the Qiagen GeneRead DNA FFPE kit. The selection of this kit was made to avoid artifacts, such as the deamination of cytosine bases to deoxyuracil introduced by fixation and embedding conditions. Removal of these artifacts was essential to obtain the optimized quality of DNA for next-generation sequencing (NGS). For the first step of DNA extraction, after adding a dewaxing solution, the cores were lysed in a tissue lyser. Qiacube was used for the last steps of DNA binding, washes and elution.

# DNA concentration was determined using the qubit fluorometer. An agreed aliquot of all 100 samples was delivered to Labs 1 and 2 for quality control and analyses.

# **Lab 1**

## ***Sequencing data analysis***

## *Samples pre-processing*

Samples were sequenced with NextSeq 500 (Illumina, USA). Raw FASTQ files were then aligned with the BWA's reference genome (hg19).^3^ Variant calling was performed with VarDict,^4^ and ploidy and purity were estimated with PureCN.^5^ Raw reads from the instrument were demultiplexed and converted from BCL (Illumina’s proprietary base calling format) to FASTQ using bcl2fastq (Illumina, USA). Reads then

underwent adapter trimming with atropos^6^ and then aligned to the reference genome with the Burrows-Wheeler Aligner (BWA).^3^ Coverage assessment was performed with mosdepth.^7^

## *Process-matched reference generation*

To generate the panel of normals (PoN), we used 70 non-tumor (blood) samples. Aligned files from non-tumor samples are genotyped with the Genome Analysis Toolkit 4.1 (GATK), using the HaplotypeCaller runner.^8^

Raw variant calls from samples were then filtered with the following, stringent, criteria:

- Quality <2.0
- Strand bias (Fisher’s test) >60 (SNPs) or >200 (indels)
- Mapping quality <30
- ReadPosRankSum <-20 (indels)
- Low quality variants (ReadPosRankSum <0) with low fraction (<25%) at the end of the reads

Variant calls, in VCF format, were then annotated using the Variant Effect Predictor (VEP)^9^ and processed with PureCN,^5^ were then combined with GC-normalized, loess-smoothed coverage from the same samples to create a panel of normals (PoN) used as a reference.

## ***Somatic variant calling and annotation***

Somatic variant calling on tumor samples was performed with VarDict,^4^ ran in tumor-only mode.

Variants were then annotated using the VEP and vcfanno.^10^ The latter adds annotations from several databases:

- dbSNP
- gnomAD
- COSMIC
- ClinVar

Lastly, variants were prioritized according on their presence in public databases.

## ***Purity and ploidy estimation***

PureCN^5^ was used to perform purity and ploidy estimation. For all tested samples, GC-normalized coverage was generated and to avoid interference from natural copy number variations, a blacklist of simple repeats across the genome (obtained from the UCSC Genome Browser^11^) was supplied during the analysis.

Lastly, internal segmentation is carried out with a patched version of PSCBS.^12,13^

The entire procedure ran on a widely-available open-source pipeline (“bcbio”^14^) running on a high-performance computing (HPC) cluster.

##

## ***Variant post-processing***

## *Collection and filtering of variants*

VCF files in output from the previous somatic variant calling workflow were assembled together, annotated and used to build a GEMINI-compatible database^15^ using an in-house developed software.

Database preparation and queries were performed with variantdb, an in-house developed Python library which queries the database using the SQLalchemy framework.^16^

Once the database is prepared, variants were filtered according to several criteria.

A first set of filters were used to remove variants with:

- A coverage lower than 60X;
- A variant allele fraction (VAF) lower than 10%;
- Low or no impact on the protein (intergenic, intronic, UTR, synonymous, start retained and stop retained variants).

While a second set of filters is applied to remove:

- Variants with a known population frequency lower than 1% in the combined data from GNOMAD and the 1000 genomes project;
- Variant clinically validated as ‘benign’ or ‘likely benign’ on ClinVar.

Variants suspected of being technical artifacts, such as variants with high frequency (higher than 60% of the considered samples, in particular frameshift or indels in poly-X regions), were removed using an in-house Python script.

Lastly, variants were validated through the Cancer Genome Interpreter (CGI) database,^17^ BRCA Exchange,^18^ COSMIC^19^ and ENIGMA^20^ using an in-house Python script.

Variants marked as *cancer drivers* on CGI were kept, while variants marked as *benign* or *unknown significance* on the other database were discarded.

Cancer drivers includes:

- **Known driver mutations** from the literature;
- **TIER 1**: strong probability of being drivers;
- **TIER 2**: medium probability of being drivers.

The dataset thus obtained, containing mutational information, is further merged with the data obtained from PureCN. This generates a combined dataset containing purity, copy number and loss of heterozygosity (LOH) information: this step is performed using another in-house Python script.

Lastly, as an HR pathway deficiency only occurs with a complete loss of one (or more) proteins that belong to the pathway, only variants flagged as having LOH in PureCN data (meaning the loss of the other, wild type allele) are kept. Sub-clonal variants (as determined in PureCN) in HR genes are also discarded.

The entire pipeline, from the assembly of the variants to filtering, was developed through Nextflow,^21^ a reactive workflow framework which allow to join different processes written in different scripting languages.

## ***HRD score calculation***

Genomic signatures are demonstrated to be predictive biomarkers of homologous recombination deficiency (HRD) in ovarian cancer.^22^ Using these genomic signatures, Telli et al.^23^ derived the HRD scores, an HRD predictive biomarker which consists of the sum of three independent measures of genomic instability reflecting structural aberrations:

1. **LOH:** the number of sub-chromosomal regions with LOH at least 15 Mbp in length^24^;
2. **LST:** Large Scale state Transitions, which represents the number of chromosomal breaks between flanking regions of at least 10 Mbp, but no further spaced than 3 Mbp^25^
3. **TAI:** the number of sub-chromosomal regions undergoing Allelic Imbalance extending to the Telomers, but not the whole chromosome.^26^

The score is thus calculated using an in-house Python script, which works as follows:

1. Pre-processing of the variants, removing flagged variants from PureCN and construct a ‘major allele CN’;
2. Variant segment generation:
   1. Group segments (generated before) by chromosome (but do only autosomes);
   2. Exclude data from chromosomes where minor CN is consistently 0 (LOH of the whole chromosome);
   3. Set all segments with major allele CN > 1 as equal 1 (they count all the same);
   4. Re-aggregate the segments (see point 3 of Segment generation);
   5. Select those segments where major CN is 1 and minor CN is 0;
   6. Select those segments where the length of the segments is at least 15 Mbp;
   7. Sum the number of segments which are left after the selection (see point 6) in that chromosome to the LOH score.
3. LOH, TAI and LST evaluation.

#### LOH score

The LOH score is calculated as follows:

1. Group segments (those generated before) by chromosome (but do only autosomes);
2. Exclude data from chromosomes where minor CN is consistently 0 (LOH of the whole chromosome);
3. Set all segments with major allele CN > 1 as equal 1 (they count all the same);
4. Re-aggregate the segments (see point 3 of Segment generation);
5. Select those segments where major CN is 1 and minor CN is 0;
6. Select those segments where the length of the segments is at least 15 Mbp;
7. Sum the number of segments wihch are left after the selection in 6. in that chromosome to the LOH score;

#### LST score (large scale transitions)

Large scale transitions are calculated as follows:

1. Group segments (those generated before) by chromosome (only on autosomes);
2. Subset the data for those segments that wholly fall into p or q arm and not those across;
3. Handle p and q data separately (because the following steps change start or end depending on the arm);
4. Re-aggregate the segments (see point 3 of Variant segment generation) for each arm;
5. Set the last (p arm) or the first (q arm) start position as the corresponding centromere coordinate (start and end, respectively);
6. Recompute segment lengths;
7. Identify all segments that are below 3 Mbp;
8. Iterate through these segments:
   1. Remove the segment from the data (and from the list at point 7);
   2. Re-aggregate the segments (see point 3 of Variant segment generation);
   3. Recompute segment lengths;
   4. Repeat from step 1 until all the segments <3 Mbp have been removed;
9. Select all pairs of adjacent segments that are ≥10 Mbp in length and spaced less than 3 Mbp;
10. Count these segments and add the value to the LST score;

#### TAI (Telomeric allelic imbalance)

1. Group segments (those generated before) by chromosome (but only autosomes; the following operations are applied to each group);
2. Discard all segments smaller than 1 Mbp (to reduce noise);
3. Re-aggregate the segments (see point 3 of Variant segment generation);
4. If there is only one segment covering the entire chromosome, pass to the next chromosome;
5. Get the absolute minimum major copy number observed in the segments for that chromosome;
6. For each segment, consider the major and minor CN:
   1. If the minimum major CN is equal to 1 or the minimum major CN has an even number of copies, check if there is a difference between major and minor CN: if there is, mark the segment has having “Interstitial” allelic imbalance;
   2. If the condition at point 1 is *not* satisfied, we have an odd number of copies of major allele copy number; consider the contribution of the minor allele to the imbalance: if the minor allele copy number in that segment is not 0 and the sum of major and minor allele copy number for that segment is equal to the minimum copy number observed, mark the segment as having “Interstitial” allelic imbalance;
   3. If neither 1 or 2 holds, mark the segment as having no imbalance;
7. Get the tagged segment from point 6 closest to the 5’ telomere and the segment closest to the 3’ telomere:
   1. If the segment closest to the 5’ telomere ends before the centromere, mark the segment as having “telomeric allelic imbalance” and add 1 to the TAI score;
   2. If the segment closest to the 3’ telomere starts before the centromere, mark the segment as having “telomeric allelic imbalance” and add 1 to the TAI score.

**Lab 2**

***Library preparation***

DNA libraries for Illumina sequencing were prepared using the KAPA HyperPlus kit (Roche Sequencing Solutions, Pleasanton, CA, USA). The library preparation was performed using a concentration of extracted DNA to 50 ng/µl according to the manufacturer’s protocol except for the following modified steps: to achieve an average DNA fragment size between 180 and 220 bp we performed an enzymatic fragmentation for 30 minutes at 37°C; the ligation reaction was incubated at 20°C for 1 hour. Finally, the quality and integrity of libraries were assessed on the TapeStation (Agilent Technologies, Santa Clara, CA, USA). The concentration of all libraries was measured using Qubit dsDNA High Sensitivity (HS) assay kit on Qubit® Fluorometer 4.0 (Invitrogen Co., Life Sciences, CA, USA) after an equimolar pool was prepared. The sequencing reaction was carried out on the Illumina NextSeq550 Dx System (Illumina, San Diego, CA, USA), loading the pool with a concentration of 1.2 pM and 2% Phix 1.5 pM.

## ***Sequencing data analysis***

Two different NextSeq500/550 Mid output kits (300 cycles) were loaded on NextSeq550 Dx in RUO mode to sequence the training set (n=24 samples). Eight samples were analyzed in the first run and 16 samples in the second, including five analytical duplicates from the previous one. The test set (n=100) was divided into 50 samples without duplicates on two independent NextSeq500/550 high-output kits (300 cycles). For each run, quality of the sequenced samples was checked using MulitQC software. Fastq files were then aligned to the hg19 reference genome using BWA-MEM. Moreover, supplementary and duplicate reads were removed from the BAM files using Samtools and PicardTools' MarkDuplicates, respectively. An overall view of the sequencing alignment data was performed at this stage to detect biases in the data's sequencing and/or mapping. To check the aligned BAM files, we used ‘Multi Sample BAM QC’ option available in the platform-independent tool Qualimap v2.2.1. All the BAM files that have met quality criteria were sent to downstream analysis. We used the DNAseq R package to divide sequencing genome data into non-overlapping fixed-sized bins to estimate chromosomal aberrations in our samples. The number of reads in each bin is then counted and corrected for sequence mappability and GC content. Finally, reads were filtered to remove spurious regions in the genome.

***BRCA testing***

To investigate the tumor samples' BRCA status (HRR assessment), the TruSight™ Tumor 170 kit (namely TS170; Illumina, San Diego, CA, USA) was used. This NGS assay is designed to cover a wide range of genes and variant types associated with solid tumors targeting DNA variants from formalin-fixed, paraffin-embedded (FFPE) tumor samples. Libraries for TS170 were prepared using 50 ng of gDNA, performing the following steps. gDNA was fragmented to about 250 bp size using the Covaris M220 Focused-ultrasonicator and microTUBE-50 AFA Fiber Screw-Cap (Covaris, Woburn, MA) with the following settings: peak incident power 75 watts, duty factor 15%, 1000 cycles per burst, 360 seconds treatment time, 20°C temperature.

According to the TS170 protocol, the regions of interest are hybridized to biotinylated probes, magnetically pulled down with streptavidin-coated beads, and eluted to enrich the library pool. Finally, the libraries are normalized using a simple bead-based protocol before pooling and sequencing. The sequencing reaction was carried out on the Illumina NextSeq550 Dx System (Illumina), loading the pool with a concentration of 1.8 pM and 2% Phix 1,5 pM using a High Output flow cell kit (Illumina).

***DRY lab***

The analysis on high-grade serum carcinoma samples was performed with the Clinical Genomic Workspace (CGW) Pierian DX IVD pipeline with default settings for IV diagnostics.

The software classifies all variants per the AMP classification system into tiers IA, IB, IIC, IID, III and IV. These tiers are stratified by clinical utility ('actionability' for clinical decision-making as to diagnosis, prognosis, treatment options, and carrier status) and previously reported data in the medical literature. Variations found in gnomAD^27^ that have ≥1% minor allele frequency (except those that are also in Clinvar denoted as clinically relevant, used in a clinical diagnostic assay, or reported as a mutation in a publication) are classified as known polymorphisms. Moreover, all DNA variants (SNVs, Insertions, Deletions, MNVs, and CNVs) are included only if the minimum variant allele frequency is higher than 5%. If they had a coverage depth of ≥100x (SNVs) and ≥250x (insertions, deletions and MNVs) to reliably call small DNA variants (SNVs, insertions, deletions and MNVs).

**Lab 3**

***Immunofluorescence primary and secondary antibodies***

Rabbit anti-RAD51 (Abcam ab133534, 1:1000), mouse anti-geminin (NovoCastra NCL-L, 1:100 in PDX samples, 1:60 in patient samples), rabbit anti-geminin (ProteinTech 10802-1-AP, 1:400), mouse anti-phospho-γH2AX (Millipore #05-636, 1:200). Goat anti-rabbit Alexa fluor 568 (Invitrogen; 1:500), goat anti-mouse Alexa fluor 488 (Invitrogen; 1:500), donkey anti-mouse Alexa fluor 568 (Invitrogen; 1:500), and goat anti-rabbit Alexa fluor 488 (Invitrogen; 1:500) were used as secondary antibodies.

**References**

1. Daniele G, Raspagliesi F, Scambia G, Pisano C, Colombo N, Frezzini S, et al. Bevacizumab, carboplatin, and paclitaxel in the first line treatment of advanced ovarian cancer patients: the phase IV MITO-16A/MaNGO-OV2A study. Int J Gynecol Cancer. 2021;31(6):875-882. doi: 10.1136/ijgc-2021-002434.
2. Califano D, Russo D, Scognamiglio G, Losito NS, Spina A, Bello AM, et al. Ovarian cancer translational activity of the Multicenter Italian Trial in Ovarian Cancer (MITO) group: lessons learned in 10 years of experience. Cells. 2020;9(4):903. doi: 10.3390/cells9040903.
3. Li H. (2013) Aligning sequence reads, clone sequences and assembly contigs with BWA-MEM. arXiv:1303.3997v2.
4. Lai Z, Markovets A, Ahdesmaki M, Chapman B, Hofmann O, McEwen R, Johnson J, Dougherty B, Barrett JC, and Dry JR. VarDict: a novel and versatile variant caller for next-generation sequencing in cancer research. Nucleic Acids Res. 2016, pii: gkw227.
5. Riester M, Singh A, Brannon A, Yu K, Campbell C, Chiang D, Morrissey M). PureCN: Copy number calling and SNV classification using targeted short read sequencing. Source Code for Biology and Medicine 2016;11:13. doi: 10.1186/s13029-016-0060-z.
6. Didion JP, Martin M, Collins FS. Atropos: specific, sensitive, and speedy trimming of sequencing reads. PeerJ 2017;5:e3720. https://doi.org/10.7717/peerj.3720
7. Pedersen BS, Quinlan AR. Mosdepth: quick coverage calculation for genomes and exomes. Bioinformatics. 2018;34(5):867-868. doi: 10.1093/bioinformatics/btx699.
8. Poplin R, Ruano-Rubio V, DePristo MA, et al. Scaling accurate genetic variant discovery to tens of thousands of samples. bioRxiv; 2017. DOI: 10.1101/201178.
9. McLaren W, Gil L, Hunt SE, Riat HS, Ritchie GR, Thormann A, Flicek P, Cunningham F. The Ensembl Variant Effect Predictor. Genome Biol. 2016;17(1):122. doi: 10.1186/s13059-016-0974-4.
10. Pedersen BS, Layer RM, Quinlan AR. Vcfanno: fast, flexible annotation of genetic variants. Genome Biol 2016;17:118. https://doi.org/10.1186/s13059-016-0973-5
11. <https://genome.ucsc.edu>
12. Olshen AB, Bengtsson H, Neuvial P, Spellman PT, Olshen RA, Seshan VE. Parent-specific copy number in paired tumor-normal studies using circular binary segmentation. Bioinformatics. 2011;27(15):2038-46. doi: 10.1093/bioinformatics/btr329. Epub 2011 Jun 11.
13. <https://github.com/HenrikBengtsson/PSCBS/pull/49>
14. <https://bcbio-nextgen.readthedocs.io/en/latest/index.html>
15. Paila U, Chapman BA, Kirchner R, Quinlan AR. GEMINI: Integrative Exploration of Genetic Variation and Genome Annotations. PLOS Computational Biology 2013;9(7):e1003153.
16. Michael Bayer. SQLAlchemy. In Amy Brown and Greg Wilson, editors, The Architecture of Open Source Applications Volume II: Structure, Scale, and a Few More Fearless Hacks 2012 http://aosabook.org
17. Tamborero D, Rubio-Perez C, Deu-Pons J, Schroeder MP, Vivancos A, Rovira A, Tusquets I, Albanell J, Rodon J, Tabernero J, de Torres C, Dienstmann R, Gonzalez-Perez A, Lopez-Bigas N. Cancer Genome Interpreter annotates the biological and clinical relevance of tumor alterations, bioRxiv 140475; doi: <https://doi.org/10.1101/140475>
18. Cline MS, Liao RG, Parsons MT, Paten B, Alquaddoomi F, Antoniou A, et al. BRCA Challenge: BRCA Exchange as a global resource for variants in *BRCA1*and *BRCA2*. PLoS Genet 2018;14(12):e1007752. <https://doi.org/10.1371/journal.pgen.1007752>
19. Tate JG, Bamford S, Jubb HC, Sondka Z, Beare DM, Bindal N, Boutselakis H, Cole CG, Creatore C, Dawson E, Fish P, Harsha B, Hathaway C, Jupe SC, Kok CY, Noble K, Ponting L, Ramshaw CC, Rye CE, Speedy HE, Stefancsik R, Thompson SL, Wang S, Ward S, Campbell PJ, Forbes SA. COSMIC: the Catalogue Of Somatic Mutations In Cancer. Nucleic Acids Res. 2019;47(D1):D941-D947. doi: 10.1093/nar/gky1015.
20. Thompson PM, Stein JL, Medland SE, Hibar DP, Vasquez AA, Renteria ME, Toro R, Jahanshad N, Schumann G, Franke B, Wright MJ, Martin NG, Agartz I, Alda M, Alhusaini S, Almasy L, Almeida J, Alpert K, Andreasen NC, Andreassen OA, Apostolova LG, Appel K, Armstrong NJ, Aribisala B, Bastin ME, Bauer M, Bearden CE, Bergmann O, Binder EB, Blangero J, Bockholt HJ, Bøen E, Bois C, Boomsma DI, Booth T, Bowman IJ, Bralten J, Brouwer RM, Brunner HG, Brohawn DG, Buckner RL, Buitelaar J, Bulayeva K, Bustillo JR, Calhoun VD, Cannon DM, Cantor RM, Carless MA, Caseras X, Cavalleri GL, Chakravarty MM, Chang KD, Ching CR, Christoforou A, Cichon S, Clark VP, Conrod P, Coppola G, Crespo-Facorro B, Curran JE, Czisch M, Deary IJ, de Geus EJ, den Braber A, Delvecchio G, Depondt C, de Haan L, de Zubicaray GI, Dima D, Dimitrova R, Djurovic S, Dong H, Donohoe G, Duggirala R, Dyer TD, Ehrlich S, Ekman CJ, Elvsåshagen T, Emsell L, Erk S, Espeseth T, Fagerness J, Fears S, Fedko I, Fernández G, Fisher SE, Foroud T, Fox PT, Francks C, Frangou S, Frey EM, Frodl T, Frouin V, Garavan H, Giddaluru S, Glahn DC, Godlewska B, Goldstein RZ, Gollub RL, Grabe HJ, Grimm O, Gruber O, Guadalupe T, Gur RE, Gur RC, Göring HH, Hagenaars S, Hajek T, Hall GB, Hall J, Hardy J, Hartman CA, Hass J, Hatton SN, Haukvik UK, Hegenscheid K, Heinz A, Hickie IB, Ho BC, Hoehn D, Hoekstra PJ, Hollinshead M, Holmes AJ, Homuth G, Hoogman M, Hong LE, Hosten N, Hottenga JJ, Hulshoff Pol HE, Hwang KS, Jack CR Jr, Jenkinson M, Johnston C, Jönsson EG, Kahn RS, Kasperaviciute D, Kelly S, Kim S, Kochunov P, Koenders L, Krämer B, Kwok JB, Lagopoulos J, Laje G, Landen M, Landman BA, Lauriello J, Lawrie SM, Lee PH, Le Hellard S, Lemaître H, Leonardo CD, Li CS, Liberg B, Liewald DC, Liu X, Lopez LM, Loth E, Lourdusamy A, Luciano M, Macciardi F, Machielsen MW, Macqueen GM, Malt UF, Mandl R, Manoach DS, Martinot JL, Matarin M, Mather KA, Mattheisen M, Mattingsdal M, Meyer-Lindenberg A, McDonald C, McIntosh AM, McMahon FJ, McMahon KL, Meisenzahl E, Melle I, Milaneschi Y, Mohnke S, Montgomery GW, Morris DW, Moses EK, Mueller BA, Muñoz Maniega S, Mühleisen TW, Müller-Myhsok B, Mwangi B, Nauck M, Nho K, Nichols TE, Nilsson LG, Nugent AC, Nyberg L, Olvera RL, Oosterlaan J, Ophoff RA, Pandolfo M, Papalampropoulou-Tsiridou M, Papmeyer M, Paus T, Pausova Z, Pearlson GD, Penninx BW, Peterson CP, Pfennig A, Phillips M, Pike GB, Poline JB, Potkin SG, Pütz B, Ramasamy A, Rasmussen J, Rietschel M, Rijpkema M, Risacher SL, Roffman JL, Roiz-Santiañez R, Romanczuk-Seiferth N, Rose EJ, Royle NA, Rujescu D, Ryten M, Sachdev PS, Salami A, Satterthwaite TD, Savitz J, Saykin AJ, Scanlon C, Schmaal L, Schnack HG, Schork AJ, Schulz SC, Schür R, Seidman L, Shen L, Shoemaker JM, Simmons A, Sisodiya SM, Smith C, Smoller JW, Soares JC, Sponheim SR, Sprooten E, Starr JM, Steen VM, Strakowski S, Strike L, Sussmann J, Sämann PG, Teumer A, Toga AW, Tordesillas-Gutierrez D, Trabzuni D, Trost S, Turner J, Van den Heuvel M, van der Wee NJ, van Eijk K, van Erp TG, van Haren NE, van 't Ent D, van Tol MJ, Valdés Hernández MC, Veltman DJ, Versace A, Völzke H, Walker R, Walter H, Wang L, Wardlaw JM, Weale ME, Weiner MW, Wen W, Westlye LT, Whalley HC, Whelan CD, White T, Winkler AM, Wittfeld K, Woldehawariat G, Wolf C, Zilles D, Zwiers MP, Thalamuthu A, Schofield PR, Freimer NB, Lawrence NS, Drevets W; Alzheimer’s Disease Neuroimaging Initiative, EPIGEN Consortium, IMAGEN Consortium, Saguenay Youth Study (SYS) Group. The ENIGMA Consortium: large-scale collaborative analyses of neuroimaging and genetic data. Brain Imaging Behav. 2014;8(2):153-82. doi: 10.1007/s11682-013-9269-5.
21. Di Tommaso P, Chatzou M, Floden EW, Barja PP, Palumbo E, Notredame C. Nextflow enables reproducible computational workflows. Nat Biotechnol. 2017;35(4):316-319. doi: 10.1038/nbt.3820.
22. Chen X, Schulz-Trieglaff O, Shaw R, Barnes B, Schlesinger F, Källberg M, Cox AJ, Kruglyak S, Saunders CT. Manta: rapid detection of structural variants and indels for germline and cancer sequencing applications. Bioinformatics. 2016;32(8):1220-2. doi: 10.1093/bioinformatics/btv710.
23. Vanderstichele A, Busschaert P, Olbrecht S, Lambrechts D, Vergote I. Genomic signatures as predictive biomarkers of homologous recombination deficiency in ovarian cancer. Eur J Cancer. 2017;86:5-14. doi: 10.1016/j.ejca.2017.08.029.
24. Telli ML, Timms KM, Reid J, Hennessy B, Mills GB, Jensen KC, Szallasi Z, Barry WT, Winer EP, Tung NM, Isakoff SJ, Ryan PD, Greene-Colozzi A, Gutin A, Sangale Z, Iliev D, Neff C, Abkevich V, Jones JT, Lanchbury JS, Hartman AR, Garber JE, Ford JM, Silver DP, Richardson AL. Homologous Recombination Deficiency (HRD) Score Predicts Response to Platinum-Containing Neoadjuvant Chemotherapy in Patients with Triple-Negative Breast Cancer. Clin Cancer Res. 2016;22(15):3764-73. doi: 10.1158/1078-0432.CCR-15-2477.
25. Abkevich V, Timms KM, Hennessy BT, Potter J, Carey MS, Meyer LA, Smith-McCune K, Broaddus R, Lu KH, Chen J, Tran TV, Williams D, Iliev D, Jammulapati S, FitzGerald LM, Krivak T, DeLoia JA, Gutin A, Mills GB, Lanchbury JS. Patterns of genomic loss of heterozygosity predict homologous recombination repair defects in epithelial ovarian cancer. Br J Cancer. 2012;107(10):1776-82. doi: 10.1038/bjc.2012.451.
26. Popova T, Manié E, Rieunier G, Caux-Moncoutier V, Tirapo C, Dubois T, Delattre O, Sigal-Zafrani B, Bollet M, Longy M, Houdayer C, Sastre-Garau X, Vincent-Salomon A, Stoppa-Lyonnet D, Stern MH. Ploidy and large-scale genomic instability consistently identify basal-like breast carcinomas with BRCA1/2 inactivation. Cancer Res. 2012;72(21):5454-62. doi: 10.1158/0008-5472.CAN-12-1470.
27. <https://gnomad.broadinstitute.org/>
