# Supplementary material for: Alternative academic approaches for testing homologous recombination deficiency in ovarian cancer in the MITO16A/MaNGO-OV2 trial
Source: ESMO Open. 2022 Sep 23;7(5):100585. doi: 10.1016/j.esmoop.2022.100585 (PMC9512829; doi:10.1016/j.esmoop.2022.100585)
Supplement: Supplementary Figures 1-5 [file mmc2.docx]

**Supplementary Figure 1. Workflow for the ’Standard’ and ‘LAB2’ protocols.**


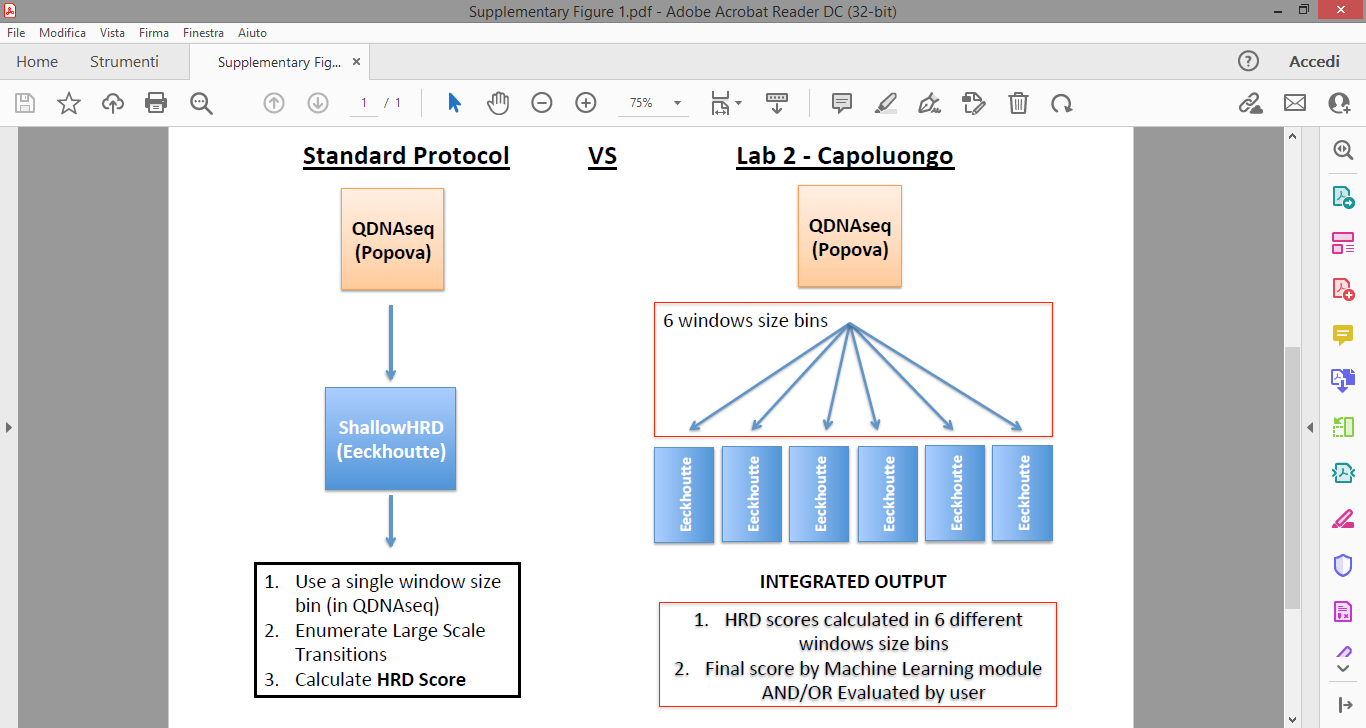


**Standard Protocol**

**LAB2 Protocol**

**Supplementary Figure 2. Study flowchart.**

**
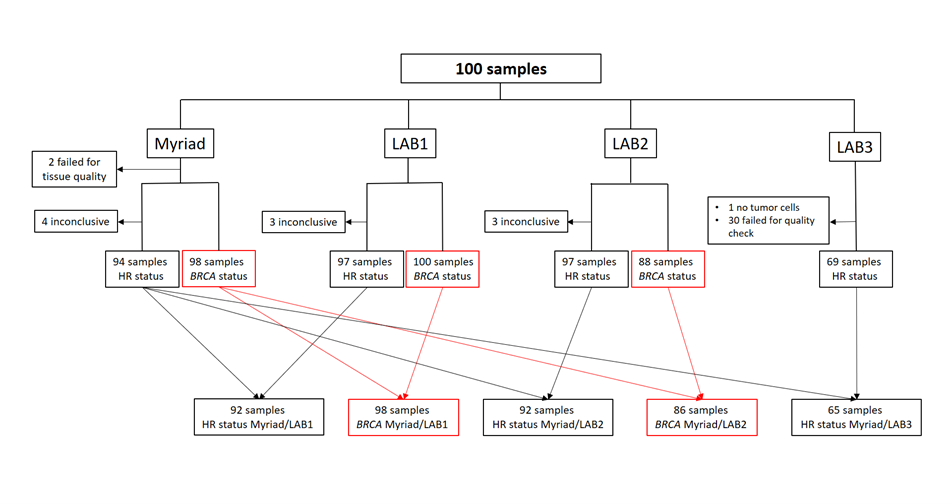
**

**Supplementary Figure 3. Progression-free survival Kaplan–Meier curves by HRR status.** A: Myriad, B: LAB1, C: LAB2, D: LAB3.


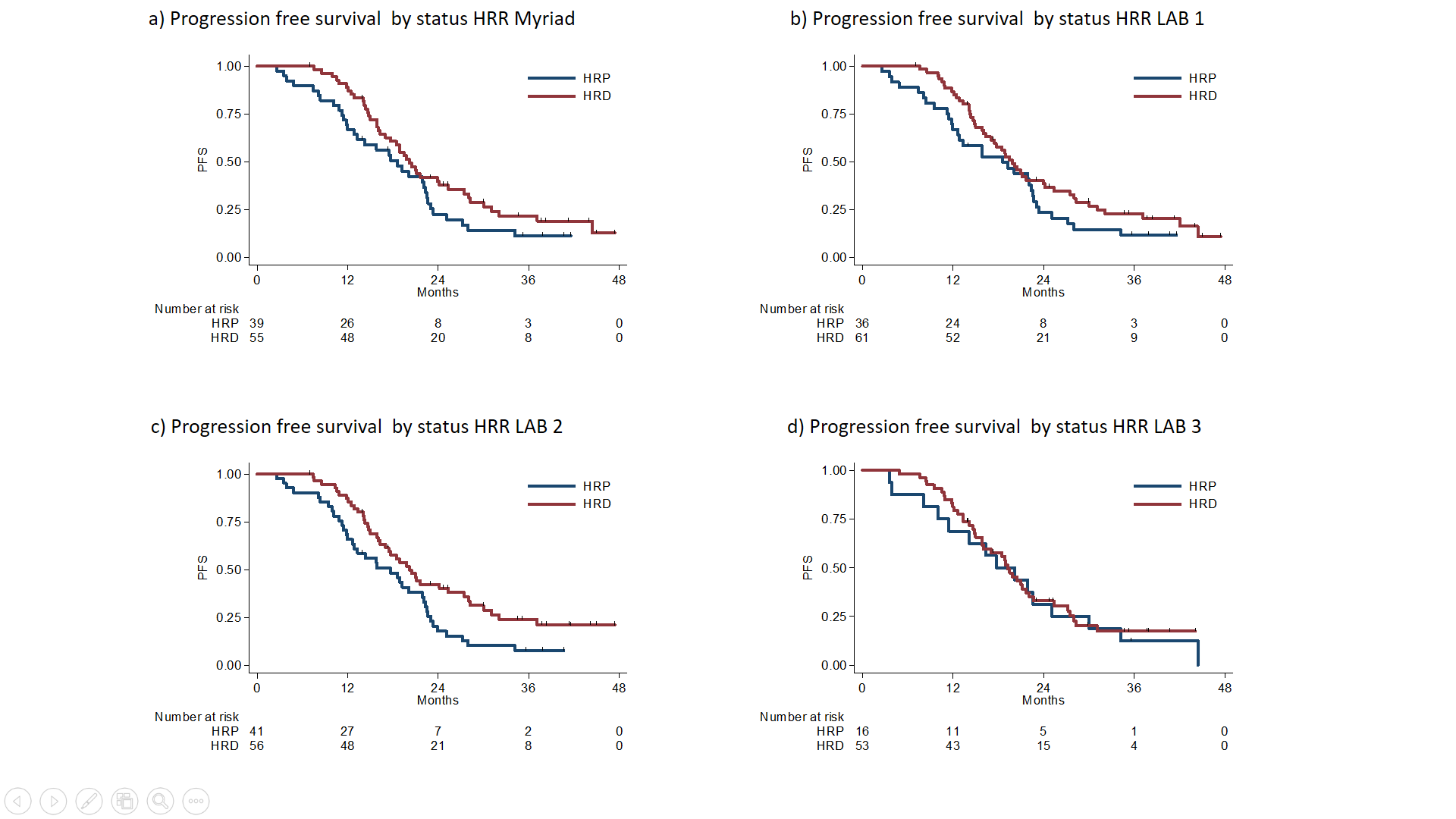


**Supplementary Figure 4. Overall survival Kaplan–Meier curves by HRR status.** A: Myriad, B: LAB1, C: LAB2, D: LAB3.


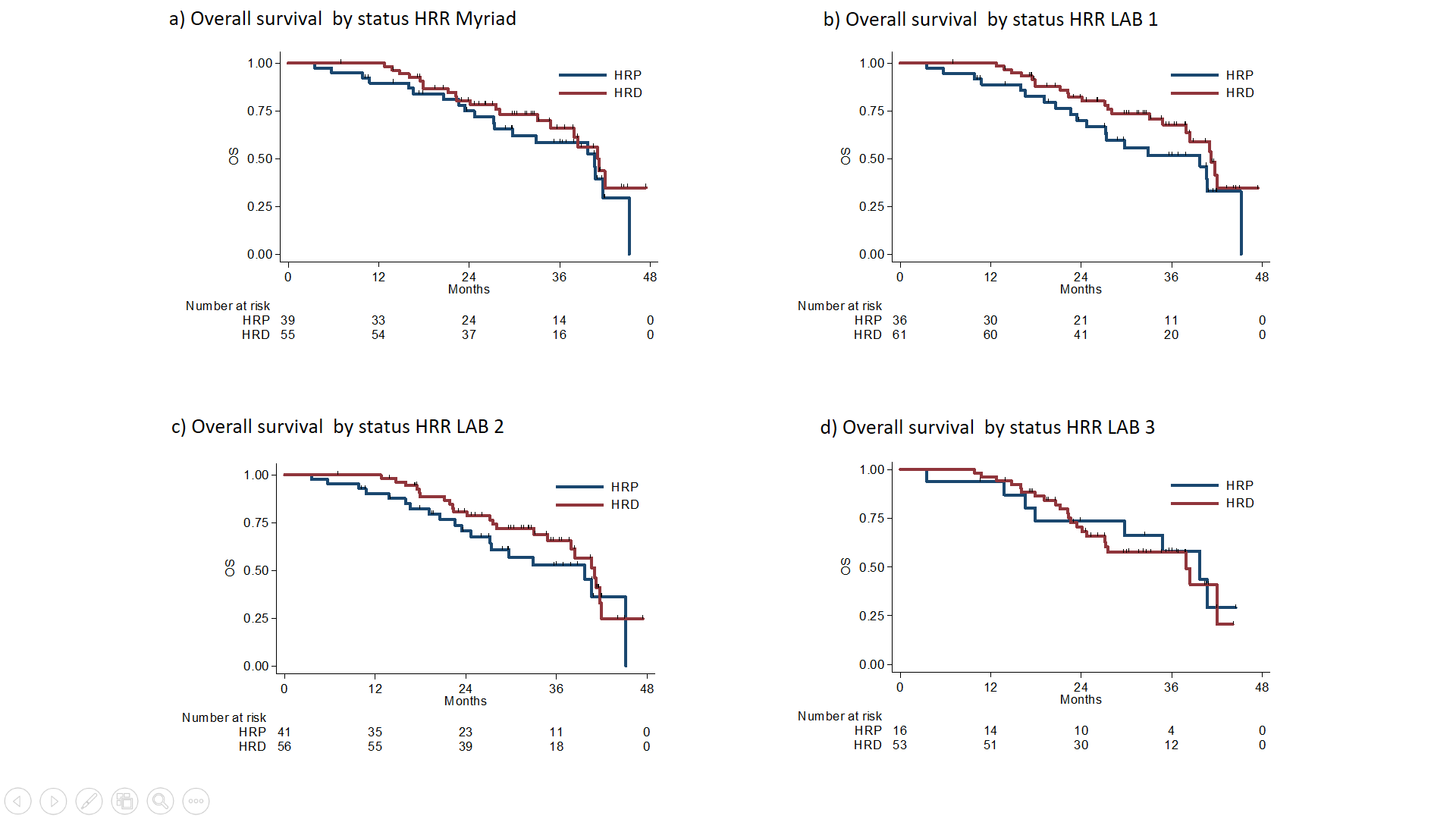


**Supplementary Figure 5. Overall progression-free survival Kaplan–Meier curves by combined Myriad and RAD51 tests.**
